# Investigation on the Effects of MXene and β-Nucleating Agent on the Crystallization Behavior of Isotactic Polypropylene

**DOI:** 10.3390/polym13172931

**Published:** 2021-08-31

**Authors:** Wanxin Peng, Jian Kang, Xiuduo Song, Yue Zhang, Bo Hu, Ya Cao, Ming Xiang

**Affiliations:** 1 State Key Laboratory of Polymer Materials Engineering, Polymer Research Institute of Sichuan University, Chengdu 610065, China; kelly_520_peng@163.com (W.P.); caoya@scu.edu.cn (Y.C.); xiangming@scu.edu.cn (M.X.); 2 Key Laboratory of Combustion and Explosion Technology, Xi’an Modern Chemistry Research Institute, Xi’an 710065, China; 3 Dongfang Electric Machinery Co., Ltd., Deyang 618000, China; metaspark@163.com (Y.Z.); nsno1@163.com (B.H.)

**Keywords:** isotactic polypropylene, MXene, β-nucleating agent, polymorphic crystallization behavior

## Abstract

The effects of MXene on the crystallization behavior of β-nucleated isotactic polypropylene (iPP) were comparatively studied. The commonly used MXene Ti_3_C_2_T_x_ was prepared by selective etching and its structure and morphology were studied in detail. Then MXene and a rare earth β-nucleating agent (NA) WBG-II were nucleated with iPP to prepare samples with different polymorphic compositions. The crystallization, melting behavior, and morphologies of neat iPP, iPP/MXene, iPP/WBG-II, and iPP/MXene/WBG-II were comparatively studied. The crystallization behavior analysis reveals that a competitive relationship exists between MXene and WBG-II when they were compounded as α and β nucleating agents. In the system, the β-nucleation efficiency (NE) of WBG-II is higher than α-NE of MXene. The β-phase has relatively low thermal stability and would transform to α-phase when cooled below a critical temperature.

## 1. Introduction

Isotactic polypropylene (iPP) is one of the most widely applied thermoplastic polymers due to its desirable properties, including good mechanical properties, excellent chemical and moisture resistance, versatile processability, and low manufacturing cost [1,2]. It is widely used in pipes, packing materials, and separators materials [3,4]. iPP is a semicrystalline polymer and exhibits polymorphic behavior [5], with a monoclinic α-form, hexagonal β-form, triclinic γ-form [6] and a mesomorphic smectic form [7]. Among the crystallization forms, the monoclinic α-form is the most common and stable one. The β-form is metastable and can only be obtained via special processing conditions such as directional crystallization in a temperature gradient [8], shear-induced crystallization [9,10], and the introduction of a selective β nucleating agent [2,11]. Among these methods, introducing a β-nucleating agent is the most effective way to access high β-phase content.

It is known that crystalline morphology and polymorphic behavior significantly affect the structure and properties of polymers. When adding a small amount of β-NA into iPP, the impact strength and toughness exceed that of α-iPP [12]. This enhanced toughness and ductility can be attributed to energy dissipation during the yield process accompanied by β to α transformation [13]. While β-iPP improves the toughness, the stiffness decreases compared with α-iPP. Thereby, compounding iPP with both α- and β- NA has become a potential way of balancing stiffness and toughness and has been widely studied. Wang et al. [14] compounded β-NA calcium pimelate with multi-walled carbon nanotubes (CNTs) and found the composites exhibited superior impact strength without a significant decrease in stiffness and strength. They attributed this improvement to the enhanced nucleating ability. Kang et al. [15] studied the crystallization behavior of CNTs compounded with β-NA WBG-II and proposed a new pathway to control the final polymorphic composition. Moreover, they found that CNTs play a leading role in the system of iPP/CNTs/WBG-II due to the higher nucleation efficiency of CNTs. The findings were also supported by the principle of determining the crystallization form of iPP nucleated with both α- and β- NA proposed by Zhong et al. [16].

MXenes are a group of 2D materials that are synthesized from ternary transition metal carbides/nitrides (MAX phase). The general formula of MXenes and MAX is M_n+1_X_n_T_x_ and M_n+1_AX_n,_ where n varies from 1 to 3, M is an early transitional metal element, X is carbon or nitrogen, T_x_ is a surface termination species such as F or/and Cl [17]. The MAX phase possesses a layered hexagonal structure in which the A-layers atoms are relatively weakly bound and act as reactive sites for removal. In 2011, the Gogotsi team firstly successively synthesized 2D Ti_3_C_2_T_x_ nanosheets by selectively etching Al atoms from a layered Ti_3_AlC_2_ ternary carbide in hydrochloric acid [18]. They called the resulting 2D materials “MXene” due to their graphene-like morphology. Since then, this discovery has led to numerous studies on the synthesis of MXenes because of the large number of MAX phase precursors available. Nowadays, over 60 MAX phases are known and about 30 different MXenes have been synthesized, and among them, Ti_3_C_2_T_x_ is the most promising material and has been studied most widely [18,19].

Like other 2D materials, the unique structure, outstanding chemical and physical properties, and wide range of stoichiometric compositions [20] of MXenes have great potential applications in many fields such as energy storage [21] and MXene-polymer composites [22]. Currently, there are a few studies on MXene-polymer composites. Ling et al. [23] firstly introduced Ti_3_C_2_T_x_ into poly(vinyl alcohol) (PVA) and poly(diallyl dimethylammonium chloride) (PDDA). They found that the composite films exhibit improved conductivities and mechanical strength and suggested that MXenes are promising fillers for multifunctional polymer composites. Zhang et al. [24] prepared Ti_3_C_2_T_x_/ultrahigh molecular weight polyethylene (UHMWPE) composites by melt mixing and found that both the thermal and mechanical properties of the composites were improved with very low Ti_3_C_2_T_x_ loading levels. The tensile and breaking strengths of the composites were enhanced and reached maximum values at 0.75 wt% Ti_3_C_2_T_x_. Shi et al. [25] fabricated Ti_3_C_2_T_x_/PP composites to improve the mechanical properties via nanoconfinement structure and physical barrier of Ti_3_C_2_T_x_ nanosheets. In their work, the results revealed that the tensile strength and ductility were improved simultaneously by 35.3% and 674.6%.

While these works focus on the effects on the physical properties of MXene/polymer composites, studies on their crystallization processes have been less reported. Cao et al. [26] prepared 2D MXene nanosheets/linear low-density polyethylene (LLDPE) by melt mixing to investigate the crystallization process. They found that 2 wt% MXene can enhance the crystallization rate of LLDPE while excessive amounts of MXene such as 4 wt% would retard the rate. Shi et al. [25] found that at low content (<1 wt%) of MXene, the crystallization temperature moved towards lower values. However, when the concentration of MXene exceeded 1 wt% the crystallization temperature started to rise. In our previous work, we studied the effects of MXene on the non-isothermal crystallization behavior of iPP [27]. When from 0 wt% to 0.5 wt% MXene was added, the crystallization peak temperature and crystallization rate both increased, indicating the crystallization process was promoted. Once the concentration of MXene reached 1 wt%, the crystallization process showed a reverse trend. Since crystallization plays a key role in the physical and mechanical properties, understanding the effects of MXene on the crystallization behaviors of polymers is of great importance. Therefore, this work chose the most common MXene, Ti_3_C_2_T_x_, and comparatively studied its effects on the crystallization and polymorphic behavior of β-iPP by differential scanning calorimetry (DSC), wide-angle X-ray diffraction (WAXD), and polarized light optical microscopy (PLOM), so as to provide new understanding of the preparation of β-iPP/MXene composites with tunable polymorphic behavior and morphologies.

## 2. Experimental Section

### 2.1. Materials

Isotactic polypropylene (iPP, trade name T38F) with average isotacticity around 97.6% and molecular weight 347,000 gmol^−1^ was provided by Lanzhou PetroChemical Corp. (Lanzhou, China). To prevent iPP from degrading, a small amount of antioxidant (Irganox 1010, BASF Corp., Ludwig, Germany) was added during the mixing process.

The β-nucleating agent (β-NA, trade name WBG-II) was supplied by Guangdong Winner Functional Materials Corp. (Foshan, China). WBG-II, a rare earth complex nucleating agent, is a commonly used commercial nucleating agent. It has a general formula Ca_x_La_1−*x*_(LIG1)_m_(LIG2)_n_, where *x* and 1 − *x* are the proportion of Ca^2+^ and La^3+^ ions, respectively, and LIG1 and LIG2 are dicarboxylic acid and amide-type ligands [13,28].

The MAX phase, Ti_3_AlC_2_ powder (99% purity, 400 mesh), was purchased from 11 Technology Co. Ltd. (Beijing, China). Concentrated hydrochloric acid (HCl, 37 wt%) was purchased from Chengdu Kelong Chemical Reagent Factory (city, China). Lithium fluoride (LiF) with 99% purity was purchased from Aladdin Bio-Chem Technology Co. Ltd. (Shanghai, China).

### 2.2. Sample Preparation

#### 2.2.1. Synthesis of Ti_3_C_2_T_x_

This work used hydrochloric acid (HCl) and LiF to form HF in situ and selectively etch Ti_3_AlC_2_. In the first step, LiF was dissolved in dilute HCl (6 M) for 5 min under magnetic stirring. Then Ti_3_AlC_2_ powder was slowly added over 10 min to avoid any overheating caused by the exothermic nature of the reaction. Then the solution was continuously heated for 24 h at 40 °C under magnetic stirring. When the etching was completed, the solution was centrifuged at 8000 rpm for 10 min and then washed with distilled water. This washing process was repeated to remove the residual reaction products until the pH of the supernatant was around 7. Then the sediment was then added with distilled water and placed in an ice-water bath followed by 2 h of ultrasonication and then centrifugation. The resulting suspension was dried under vacuum.

#### 2.2.2. Synthesis of β-iPP/Ti_3_C_2_T_x_ Composites

The samples were prepared in two steps. Firstly, the Ti_3_C_2_T_x_ and WBG-II were respectively mixed with iPP granules in a Mini-Lab Extruder (HAAKE MiniLab II, Haake Thermo Scientific, Waltham, MA, USA) at a melting temperature of 200 °C and a screw speed 60 rpm to obtain two types of master batch. Then the master batches were mixed with iPP again in a certain ratio to prepare four samples with different compositions. After that, the mixture was compressed by a pressure molding machine at 7MPa and 190 °C for further characterization. To benefit our discussion, the samples were named as iPP, iPP/WBG-II, iPP/MXene, and iPP/MXene/WBG-II, where the concentrations of Ti_3_C_2_T_x_ and WBG-II were fixed at 0.5 wt% and 0.3 wt%, respectively. The concentrations were chosen based on some previous studies in β-NA [29,30,31,32].

### 2.3. Characterization

#### 2.3.1. Differential Scanning Calorimetry

All the calorimetric experiments were conducted using a Mettler Toldeo DSC1 instrument (Mettler Corp., Zurich, Switzerland) under a nitrogen flow of 50 mL min^−^^1^. For each measurement, 2–5 mg samples were weighed and then subjected to the following standard thermal treatment: the sample was firstly heated to 200 °C and held for 5 min to erase thermal history, then cooled to end temperature of 50 °C at 10 °C/min to crystallize. Finally, it was heated back to 200 °C at 10 °C/min. The cooling and heating curves were recorded to analyze the crystallization and melting behavior of the samples. It is worth noting that to ensure the accuracy of the data, each sample was repeatedly tested five to eight times to obtain the average values.

The relative content of β-crystal (*β_c_*) can be calculated by the following equation [33,34,35]:

(1) $$\beta_{c}=\frac{{\left(1-\lambda \right)}_{\beta }}{{\left(1-\lambda \right)}_{\alpha }+{\left(1-\lambda \right)}_{\beta }}$$

where the crystallinity of each phase (1 − *λ*) is calculated by Δ*H*/Δ*H_u_*. Δ*H* and Δ*H_u_* are the melting enthalpy and the complete melting enthalpy of crystals, where the Δ*H_u_* for 100% crystalline iPP is 209 J/g [36]. Then the crystallinity of β-crystals *X_β_* is derived by multiplying the crystallinity *X_c_* and relative content of β-crystals *β_c_* [37].

#### 2.3.2. Wide-Angle X-ray Diffraction

The WAXD patterns were recorded by an Ultima IV diffractometer (Rigaku, Tokyo, Japan). A conventional Cu *K_α_* (wavelength *λ* = 0.154 nm) X-ray tube at a voltage of 40 kV and filament current of 40 mA was used to obtain the spectra in the 2*θ* range of 5–30°. The scanning rate was 10°/min and the scanning step is 0.04° [38,39]. The relative content of the β-crystal phase can be determined by the following equation [40,41]:

(2) $$k_{\beta }=\frac{H_{\beta }\left(300\right)}{H_{\beta }\left(300\right)+H_{\alpha }\left(110\right)+H_{\alpha }\left(040\right)+H_{\alpha }\left(130\right)}$$

where *k_β_* denotes the relative content of β-crystal; *H_α_* (110), *H_α_* (040), and *H_α_* (130) are the intensities of three strongest diffraction peaks of monoclinic α-form; *H_β_* (300) is the intensity of the strongest diffraction peak of hexagonal β-form.

The crystal size *L* was calculated from the WAXD data by Scherrer’s equation:

(3) L=kλβcosθ

where *β* is the diffraction line width at half maximum (FWHM) intensity in radians, *θ* is the diffraction angle at the maximum peak, and k is the shape factor and the value is taken as 0.9.

#### 2.3.3. Polarized Light Optical Microscopy

The hierarchical crystalline morphologies during crystallization were studied by polarized light optical microscopy (Eclipse LV100 POL, Nikon, Tokyo, Japan) equipped with a hot-stage (Linkam Scientific Instruments Ltd., Tadworth, UK). The sample was cut into small pieces, placed between two glass covers, and melted at 200 °C [42,43,44]. Then the sample was pressed into a thin film and slowly cooled to room temperature to allow full crystallization. The online PLOM tests were performed to observe the evolution of hierarchical crystalline morphologies. During the process, the samples were firstly heated to 200 °C at 20 °C/min for 5 min to erase thermal history, then fast cooled to 140 °C at 50 °C/min. After that, the sample was held at the temperature, and PLOM images were recorded at different times.

#### 2.3.4. Scanning Electron Microscopy

The morphology of precursor Ti_3_AlC_2_ and Ti_3_C_2_T_x_ was observed by SEM (Apreo S HiVoc, Thermo Fisher Scientific Corp., Waltham, MA, USA).

#### 2.3.5. Transmission Electron Microscope

To further investigate the structure of Ti_3_C_2_T_x_, TEM (Tecnai G2 F20 S-TWIN, FEI Corp., Hillsboro, OR, USA) was performed under an accelerating voltage of 200 kV. To prepare the TEM sample, the Ti_3_C_2_T_x_ was dispersed in distilled water under ultrasonication for 10 min, then the dispersion was dropped on the copper grid for observation.

## 3. Results and Discussions

### 3.1. Structure and Morphology of MXene

**Figure 1:**
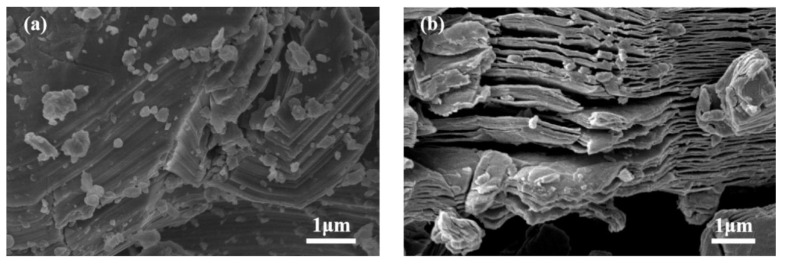
SEM images of (**a**) Ti_3_AlC_2_ and (**b**) Ti_3_C_2_T_x_ before and after etching.

The morphologies of bulk Ti_3_AlC_2_ and Ti_3_C_2_T_x_ were observed by scanning electron microscopy (SEM) as shown in Figure 1. From Figure 1a, it can be observed that the bulk Ti_3_AlC_2_ has a stacked layered structure. After the process is completed, the structure shows a relatively weak stacked structure, which can also be called an accordion-like structure. This expanded interlayer structure suggests the successive removal of Al layer and exfoliation of MXene flakes and might be caused by the escape of H_2_ gas during the exothermic reaction during the etching process [45,46].

**Figure 2:**
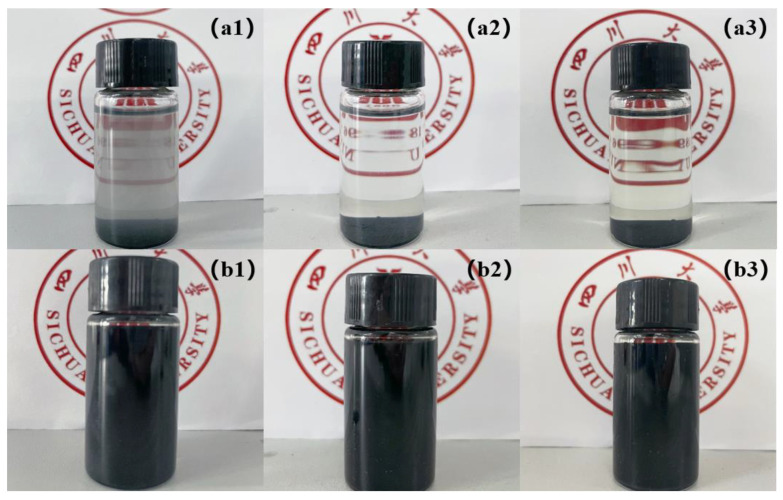
Digital photo of (**a1**–**a3**) Ti_3_AlC_2_ and (**b1**–**b3**) Ti_3_C_2_T_x_ dispersed in distilled water on day 1, day 3, and day 7.

**Figure 3:**
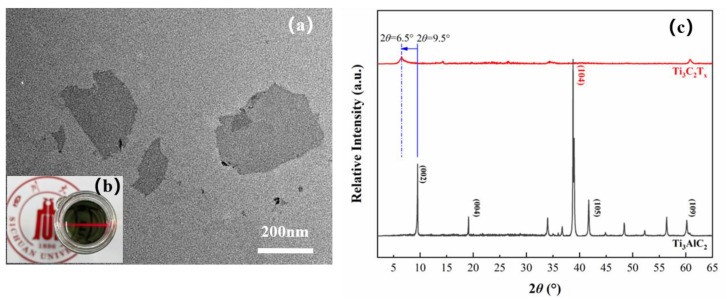
Analysis of Ti_3_C_2_T_x_ (**a**) TEM image of Ti_3_C_2_T_x_ dispersed in distilled water; (**b**) Digital photo of diluted Ti_3_C_2_T_x_ dispersion in distilled water under a red laser beam; (**c**) XRD pattern of Ti_3_AlC_2_ and Ti_3_C_2_T_x_.

Figure 2 exhibits the dispersion of Ti_3_AlC_2_ and Ti_3_C_2_T_x_ in distilled water at different times. From Figure 2a1–a3, it can be observed that the Ti_3_AlC_2_ precursor could not maintain a stable distribution in distilled water. After etching, Ti_3_C_2_T_x_ can disperse homogeneously in distilled water even after a week (Figure 2b1–b3). The digital photo in Figure 3b shows that the diluted Ti_3_C_2_T_x_ dispersion in distilled water is stable, and the Tyndall effect can be observed under a red laser beam. TEM image in Figure 3a indicates that the Ti_3_C_2_T_x_ dispersion contains single flakes with distinct edges and sizes of a few hundred nanometers. The Ti_3_C_2_T_x_ flake size can vary from a few hundred nanometers to a few microns by varying the synthesis route. There are also studies indicating that the electrical, chemical and electrochemical properties can be adjusted with respect to different lateral flake sizes, however precise control over the size still remains as a challenge [47].

The X-ray diffraction (XRD) plot in Figure 3b showed that the most intense and representative peak of Ti_3_AlC_2_ at 2*θ* ≈ 39° that is assigned to (104) plane has disappeared after etching, indicating the successful removal of Al atom in the structure [48,49]. Moreover, the (002) peak has broadened and shifted from 9.5° to 6.5°. This lower shift of (002) peak implies an increasing interlayer distance, which is consistent with the results from SEM images and is possibly caused by the removal of the Al layer in bulk structure and the introduction of surface terminations [45]. The results of SEM, TEM and XRD confirm the successful synthesis of 2D Ti_3_C_2_T_x_ MXene.

### 3.2. Crystallization Behavior of β-iPP/MXene Composites

**Figure 4:**
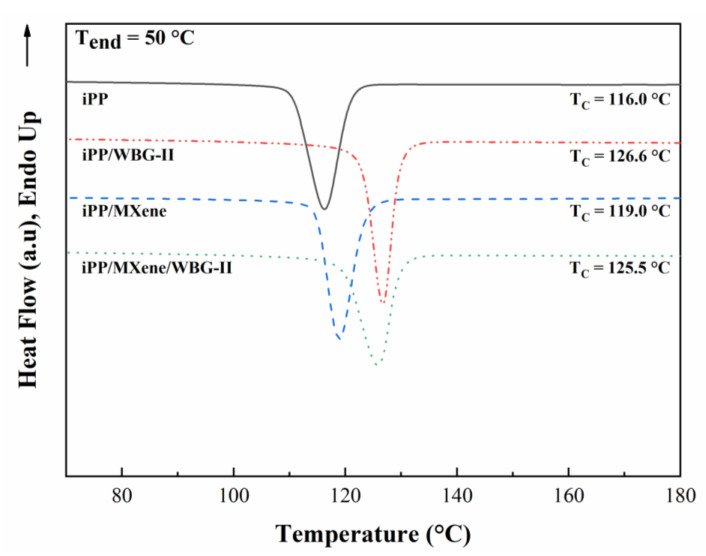
DSC cooling curve at the T_end_ = 50 °C.

**Figure 5:**
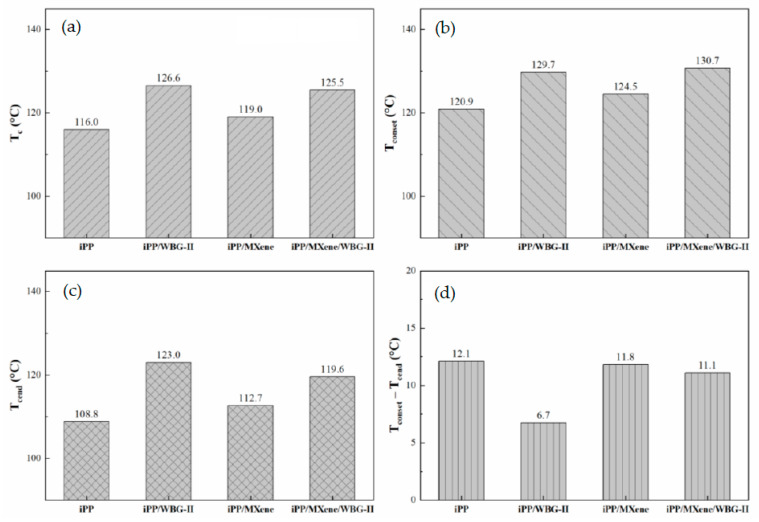
Plots of (**a**) T_c_, (**b**) T_conset_, (**c**) T_cend_, (**d**) T_conset_ − T_cend_ of four samples from the cooling curve at T_end_ = 50 °C.

To comparatively study the crystallization behavior of neat iPP, iPP/WBG-II, iPP/MXene, and iPP/MXene/WBG-II, the DSC test procedure was applied as described in the experimental section. The obtained cooling curves are shown in Figure 4. The variations in crystallization parameters including the onset, peak, end crystallization temperatures (T_conset_, T_c_, and T_cend_), and the crystallization peak width (T_conset_ − T_cend_) of different samples are plotted in Figure 5.

Figure 4 illustrates that when β-NA WBG-II is introduced into iPP, the crystallization peak temperature of iPP/WBG-II improves by 10.6 °C, and a strong β-form melting peak can be observed between the temperature range of 150–155 °C as revealed in the next section. Moreover, when MXene is introduced, the crystallization peak shifts towards higher temperature, T_c_ of iPP/MXene increases by 3 °C, and only the existence of α-form melting peak is observed in the heating curve. In addition, Figure 5 shows that T_conset_ and T_cend_ of iPP/WBG-II and iPP/MXene also increase. This shift of crystallization peak suggests that the crystallization could take place at a higher temperature, thereby the addition of WBG-II and MXene has promoted the crystallization ability of β-form and α-form crystals, respectively. On the contrary, when WBG-II and MXene are both introduced, the crystallization temperature stops increasing but drops by 1.1 °C comparing with that of iPP/WBG-II.

**Figure 6:**
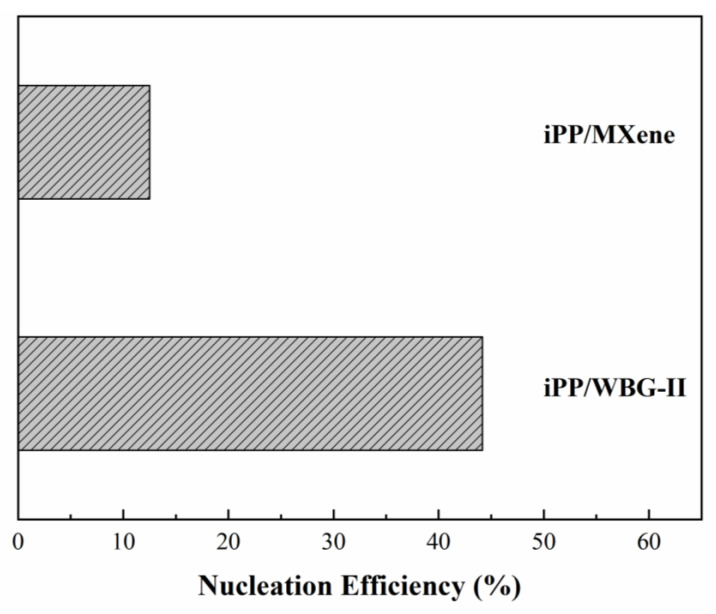
The plot of nucleation efficiencies (NEs) of iPP/WBG-II and iPP/MXene.

To further understand the variation of crystallization temperatures, the nucleation efficiency of WBG-II and MXene as nucleating agents are evaluated via the following equation developed Fillon et al. [15,50]:

(4) $$\mathrm{NE}\left(\%\right)=\frac{T_{\mathrm{cNA}}-T_{c1}}{T_{c2\max}-T_{c1}}\times 100\%$$

where T_cNA_, T_c1_, and T_cmax_ are the peak crystallization temperatures of nucleated, non-nucleated, and self-nucleated polymers. The NE is expressed as a percentage, where 100 stands for the highest efficiency and 0 stands for no nucleation behavior. Based on the work of Fillon et al. [50], the crystallization peak temperature of self-nucleated iPP is the highest and the value of T_c2max_ is taken as 140 °C. The calculated NE of WBG-II and MXene are plotted in Figure 6. It can be seen that the NE of WBG-II is evidently higher than MXene, which agrees well with the variation in T_c_. Based on the previous studies [15,16], a possible explanation in T_c_ of four samples is proposed: while both WBG-II and MXene can work as β and α nucleating agents, there exists a competitive relationship between them that would change the crystallization and polymorphic behavior of composites. According to the studies of Zhong et al. [16], WBG-II with higher NE might play the leading role in the system of iPP/MXene/WBG-II.

### 3.3. Melting Behavior of β-iPP/MXene Composites

**Figure 7:**
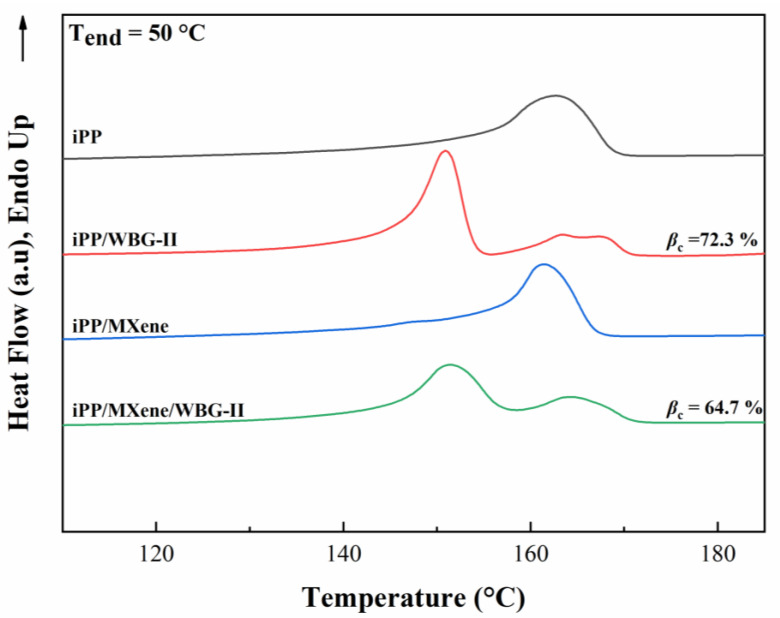
DSC heating curves of four samples.

The heating curves of four samples are plotted in Figure 7. Firstly, neat iPP and iPP/MXene only show one melting peak between 160–170 °C, which represents the α-from crystals in the structure. For iPP/WBG-II and iPP/MXene/WBG-II, two melting peaks are observed, of which the lower peak between 150–155 °C corresponds to the β-form crystals. In addition, it can be observed from the heating curves that the β-peak in iPP/WBG-II is stronger than in iPP/MXene/WBG-II. The relative content of β-crystals in two samples is calculated by Equation (1), and other parameters including peak melting temperature and degree of crystallinity are listed in Table 1. The calculated results show that the relative percentage of β-phase (*β_c_*) of iPP/WBG-II is 7.4% higher than iPP/MXene/WBG-II so that α-phase content is higher in iPP/MXene/WBG-II. Combined with the findings from crystallization curves, while both WBG-II and MXene promote the crystallization process, there might be competition between them.

**Figure 8:**
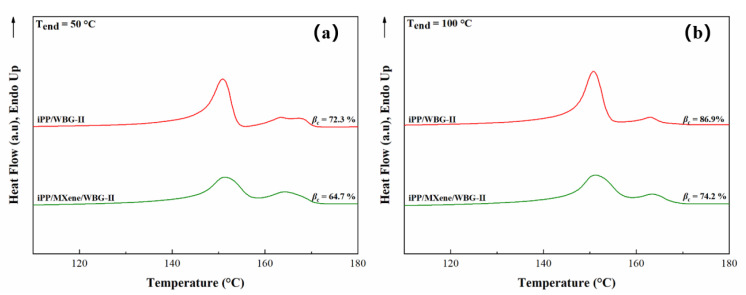
DSC heating curves of iPP/WBG-II and iPP/MXene/WBG-II at (**a**) T_end_ = 50 °C and (**b**) T_end_ = 100 °C.

Varga et al. [51,52] concluded that during the partial melting of β-crystals, β-iPP cooled down below critical temperature (T_end_ = 100–105 °C) will recrystallize into α-iPP, leading to an increase in α-phase content in the subsequent heating curve. Inversely, no βα-recrystallization will occur if the β-iPP is not cooled below the critical temperature. To comparatively study the polymorphic behavior of iPP/WBG-II and iPP/MXene/WBG-II and the thermal stability of the formed β-phase, an end temperature of cooling at T_end_ = 100 °C was also chosen to compare the heating curves at T_end_ = 50 °C. The heating curves of T_end_ = 50 °C and T_end_ = 100 °C are plotted in Figure 8a,b, and two main trends can be observed from the curves.

Firstly, for both iPP/WBG-II and iPP/MXene/WBG-II, the α-peaks at T_end_ = 50 °C appear stronger than at T_end_ = 100 °C, indicating that a certain amount of β-phase with less thermal stability transformed into α-phase during the melting process when T_end_ = 50 °C. Secondly, β-peaks of iPP/WBG-II at both end temperatures are higher than that of iPP/MXene/WBG-II. The values of *β_c_* are evaluated and listed in Figure 8. When T_end_ decreases from 100 °C to 50 °C, the *β_c_* in iPP/WBG-II and iPP/MXene/WBG-II is decreased by 14.6% and 9.5%. The decrease of *β_c_* corresponds to the β-phase with relatively low thermal stability, which transformed into α-phase via βα-recrystallization during the melting process. To be more exact, the larger the *β_c_* difference between T_end_ = 50 °C and T_end_ = 100 °C, the higher the fraction of β-phase with relatively low thermal stability. Meanwhile, the higher the *β_c_* at T_end_ = 100 °C, the stronger ability for the sample to form β-phase. Therefore, the results of Figure 7 reveal that the addition of MXene into β-iPP decreases the total amount of formed β-phase but increases the thermal stability of the formed β-phase as well, which might be attributed to the competitive effects between β-NA and MXene.

### 3.4. WAXD Analysis

**Figure 9:**
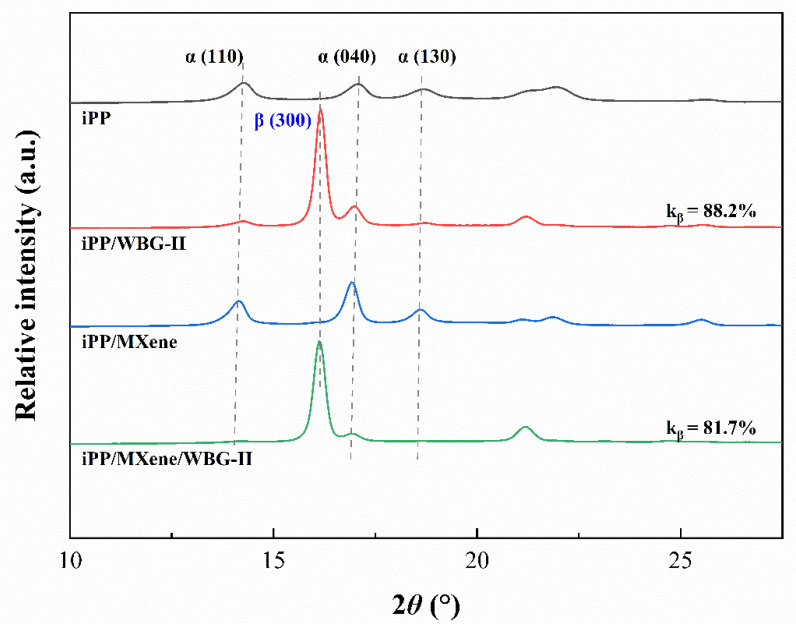
WAXD patterns of four samples.

The WAXD results of the four samples are shown in Figure 9, and the calculated crystalline structure parameters, including the 2*θ*, *d*-spacing, line width at half maximum (FWHM) in radians and crystallite sizes *L* of each peak, are listed in Table 2.

For neat iPP and iPP/MXene, the three strongest peaks are found at around 14.1°, 16.9°, and 18.6° corresponding to α (110), α (040), and α (130) diffractions of α-crystal [11,53]. For iPP/WBG-II and iPP/MXene/WBG-II, a peak around 16.1° corresponding to β (300) diffraction peak, indicating the existence of β-crystals in their structure. The relative content of β-crystal in iPP/WBG-II and iPP/MXene/WBG-II are *k_β_* = 88.2% and 81.7%, respectively. This result is in good agreement with the variation of *β_c_* in Figure 8. Both results denote that when MXene is added in β-PP, the β-phase content would decrease due to competition between α-nucleation effect of MXene and β-nucleation effect of WBG-II. Moreover, Table 2 reveals that the addition of MXene into neat iPP greatly increases the crystallite sizes *L* of the sample, while the addition of WBG-II into neat iPP decreases the *L*. When both WBG-II and MXene are added, an increase of *L* can be observed.

### 3.5. PLOM Observation

Based on the crystallization behaviors in Figure 4 and WAXD results in Figure 9, it could be observed that neat iPP and iPP/MXene show similar Tc and contain only α-phase.

Moreover, the Tc of iPP/WBG-II and iPP/MXene/WBG-II only differ by around 1 °C and they both contain β-phase. To conveniently compare the evolution of hierarchical crystalline morphologies, the samples were isothermally crystallized at the same temperature and discussed by two groups (neat iPP and iPP/MXene; iPP/WBG-II and iPP/MXene/WBG-II).

**Figure 10:**
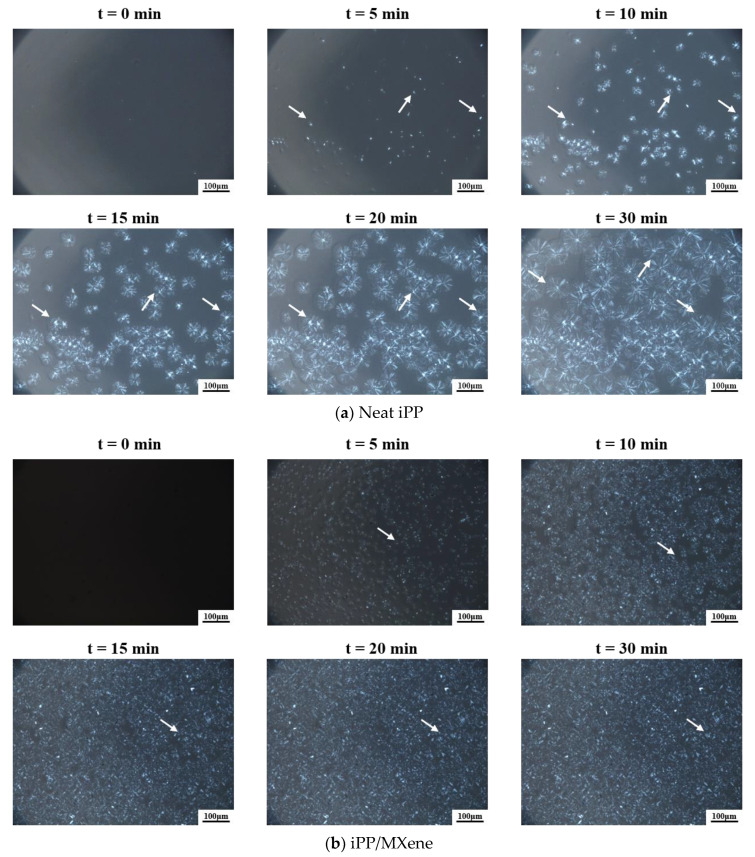

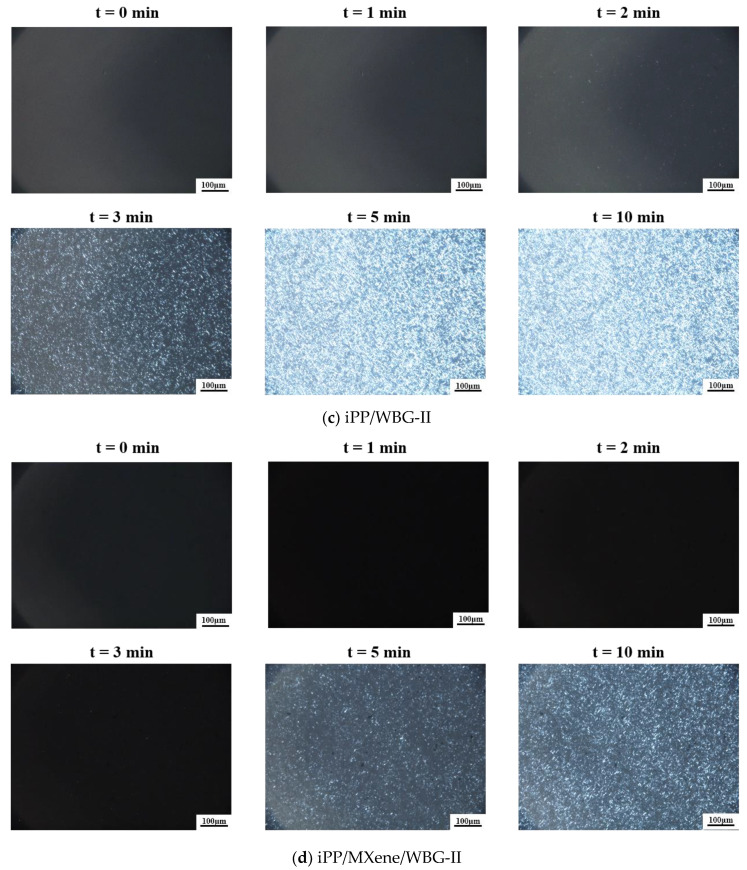
Crystallization morphological evolution during isothermal crystallization of (**a**) neat iPP (**b**) iPP/MXene (**c**) iPP/WBG-II and (**d**) iPP/MXene/WBG-II at 140 °C. The bar represents 100 μm.

From Figure 10a,b, it can be seen that α-crystal spherulites exist in iPP and iPP/MXene, but the crystallization of these two samples is quite different. For neat iPP, the crystal nuclei start to appear after 5 min from crystallization and continue to grow. After 15 min from crystallization, the number of nuclei did not increase evidently, while the spherulite size continues to increase. After 30 min, the spherulites continue to grow in size, the crystallization is yet to complete. On the contrary, for iPP/MXene, a large number of crystal nuclei occur in the field of view after 5 min from crystallization but the growth rate might be relatively slow. The PLOM images stay almost unchanged after 15 min, indicating that the crystallization is finished. Comparing iPP/MXene with neat iPP, the crystal nuclei appear much earlier with significantly higher nucleation density. However, the growth rate of nuclei seems to be slower, the crystallite size of iPP/MXene is much smaller. Under the same isothermal crystallization condition, the findings suggest that the NE of iPP/MXene is evidently higher than neat iPP, which is in accord with the DSC results above.

Figure 10c,d show that β-crystals exist in iPP/WBG-II and iPP/MXene/WBG-II. For iPP/WBG-II, the nuclei occur in the field of view 2 min after crystallization starts and continue to grow, reflecting the strong nucleation effect of WBG-II. The PLOM image stays almost unchanged but becomes brighter after 3 min, and the crystallization finishes at 5 min. To identify β-crystals from the morphology, the sample was selectively melted at 156 °C to so that the residual α-crystals could be observed [54]. The result indicates that most area of the screen is occupied with the highlighted β-crystals. However, only a few nuclei were formed after 3 min from crystallization in the case of iPP/MXene/WBG-II, indicating that iPP/WBG-II has a much higher NE at the early stage of crystallization. The PLOM image of iPP/MXene/WBG-II shows that β-crystals dominate in the morphology, which is consistent with the assumption that WBG-II plays a leading role in determining the behavior in the system. However, due to the competitive nucleation effects of WBG-II and MXene, less area of highlighted β-crystals can be seen from Figure 10d.

The PLOM images agree well with the previous DSC and WAXD analysis. When MXene is introduced into iPP, the crystallization process is promoted which might be caused by more nucleation sites. On the contrary, the introduction of MXene into iPP/WBG-II slows down the crystallization process due to the competition between MXene and WBG-II, and greatly changes the polymorphic behavior of the composites.

## 4. Conclusions

In this work, iPP was mixed with a commonly used MXene, Ti_3_C_2_T_x_, and the β-nucleating agent WBG-II to prepare samples with different compositions. The effects of MXene on the crystallization and polymorphic behavior of β-nucleated iPP were then comparatively studied. The DSC cooling curves indicated that MXene and WBG-II promote the crystallization ability of α- and β- form crystals, respectively, but the nucleation efficiency of WBG-II is higher. DSC heating curves and WAXD results showed that β-phase content in iPP/WBG-II is higher than iPP/MXene/WBG-II. These results suggest that a competitive relationship exists between MXene and WBG-II as α and β nucleating agents, and WBG-II plays a leading role in determining the crystallization of iPP nucleated with both MXene and WBG-II. To investigate the thermal stability of β-phase, β-iPP samples were cooled to two end temperatures (50 °C and 100 °C), and the resulting heating curves were compared. The results indicated that the β-phase is thermally metastable and would transform to the α phase when cooling below a critical temperature. The online PLOM results agree well with others indicating that both MXene and WBG-II improves the crystallization process of iPP and WBG-II dominates in the crystalline morphology of iPP.

## Figures and Tables

**Table 1:** The heating curve parameters of four samples, where T_m1_ and T_m2_ are the peak melting temperature of the α- and β-phase, *X_c_* is the degree of crystallinity, *α_c_* and *β_c_* are the relative percentage of α- and β-crystals, *X_α_* and *X_β_* are the crystallinity of α- and β-crystals.

Sample	T_m1_ (°C)	T_m2_ (°C)	*X_c_* (%)	*α_c_* (%)	*β_c_* (%)	*X_α_* (%)	*X_β_* (%)
iPP	162.8	—	41.4	—	—	—	—
iPP/WBG-II	163.4	150.9	44.6	27.7	72.3	12.4	20.0
iPP/MXene	161.9	—	43.0	—	—	—	—
iPP/MXene/WBG-II	164.7	151.4	41.4	35.3	64.7	14.6	22.8

**Table 2:** WAXD parameters of four samples.

Parameters	Sample	(*h k l*)			
		α (110)	α (040)	α (130)	β (300)
2*θ* (°)	iPP	14.2	17.1	18.6	—
	iPP/WBG-II	14.3	16.9	18.6	16.1
	iPP/MXene	14.1	16.9	18.6	—
	iPP/MXene/WBG-II	14.3	17.0	18.7	16.2
*d*-spacing (Å)	iPP	6.2	5.2	4.8	—
	iPP/WBG-II	6.2	5.2	4.8	5.5
	iPP/MXene	6.3	5.2	4.8	—
	iPP/MXene/WBG-II	6.2	5.2	4.7	5.5
FWHM	iPP	0.7	0.6	0.9	—
	iPP/WBG-II	0.7	0.7	1.2	0.3
	iPP/MXene	0.5	0.4	0.5	—
	iPP/MXene/WBG-II	0.6	0.5	0.5	0.3
*L* (nm)	iPP	12.1	12.7	8.5	—
	iPP/WBG-II	11.1	12.2	6.6	24.5
	iPP/MXene	16.1	20.5	17.7	—
	iPP/MXene/WBG-II	13.1	15.1	15.6	27.1

## Data Availability

Not applicable.
